# Local treatment vs. years of life lost in intermediate-risk prostate cancer

**DOI:** 10.1007/s00345-026-06858-4

**Published:** 2026-10-30

**Authors:** Fabian Falkenbach, Mattia Longoni, Andrea Marmiroli, Quynh Chi Le, Calogero Catanzaro, Michele Nicolazzini, Federico Polverino, Jordan A. Goyal, Riccardo Schiavina, Carlotta Palumbo, Gennaro Musi, Felix K. H. Chun, Alberto Briganti, Fred Saad, Shahrokh F. Shariat, Lars Budäus, Markus Graefen, Pierre I. Karakiewicz

**Affiliations:** 1 Cancer Prognostics and Health Outcomes Unit, Division of Urology, University of Montreal Health Center, Montreal, QC Canada; 2 Martini-Klinik Prostate Cancer Center, University Medical Center Hamburg-Eppendorf, Hamburg, Germany; 3 Department of Urology, University Hospital Heidelberg, Heidelberg, Germany; 4 Vita-Salute San Raffaele University, Milan, Italy; 5 Department of Urology, IEO European Institute of Oncology, IRCCS, Milan, Italy; 6 Department of Oncology and Haemato-Oncology, Università degli Studi di Milano, Milan, Italy; 7 Department of Urology, Goethe University Frankfurt, University Hospital, Frankfurt am Main, Germany; 8 Department of Urology, University of Bologna, St. Orsola-Malpighi Hospital, Bologna, Italy; 9 Division of Urology, Department of Translational Medicine, University of Eastern Piedmont, Maggiore della Carità Hospital, Novara, Italy; 10 Division of Urology, Department of Oncology, University of Turin, Orbassano, Italy; 11 Department of Neurosciences, Science of Reproduction and Odontostomatology, University of Naples Federico II, Naples, Italy; 12 Department of Urology, Comprehensive Cancer Center, Medical University of Vienna, Vienna, Austria; 13 Department of Urology, Weill Cornell Medical College, New York, NY USA; 14 Department of Urology, University of Texas Southwestern Medical Center, Dallas, TX USA; 15 Hourani Center for Applied Scientific Research, Al-Ahliyya Amman University, Amman, Jordan; 16 Department of Urology, University Medical Center Hamburg-Eppendorf, Hamburg, Germany

**Keywords:** Life expectancy, Prostate cancer, Radical prostatectomy, Radiotherapy, SEER

## Abstract

**Purpose:**

The role of radical prostatectomy (RP) or radiotherapy (RT) in intermediate-risk prostate cancer (PCa) is still under debate. Our aim was to quantify the effect of RP and RT on the average years of life lost (YLL) in patients with intermediate-risk PCa.

**Methods:**

Within the SEER database 2004–2021, intermediate-risk PCa patients aged 45–75 were identified. Stratification rested on local therapy type: RP vs. RT vs. none. We performed Monte Carlo simulation to generate individual survival estimates based on Social Security Administration life tables. Subsequently, YLL was compared between PCa patients and matched population controls.

**Results:**

Of 220,113 intermediate-risk PCa patients, 94,452 (42.9%), 85,232 (38.7%), and 40,429 (18.4%) underwent RP, RT, and no local therapy, respectively. Compared to population controls, RP and RT patients did not exhibit YLL disadvantages. Conversely, no local therapy patients exhibited a survival disadvantage of 0.21 YLL (*p* < 0.001). In the subgroup of patients diagnosed at age 45–55, a YLL disadvantage was seen for no local therapy (1.32) and RT patients (0.69), while their RP counterparts did not exhibit a YLL disadvantage. In further subgroup analyses, a substantial YLL disadvantage was recorded in unfavorable intermediate-risk PCa patients without local therapy (0.47 YLL), whereas no YLL disadvantage was seen in unfavorable intermediate-risk PCa patients who underwent RP or RT.

**Conclusion:**

In intermediate-risk PCa, no YLL disadvantage was observed in RP and RT patients within all subgroups. Patients diagnosed at a young age and with unfavorable cancer characteristics are at increased risk for premature death if not locally treated.

**Trial registration:**

doi10.17605 OSF.IO/D2RUV.

**Supplementary Information:**

The online version contains supplementary material available at https://doi.org/10.1007/s00345-026-06858-4.

## Introduction

The best initial local therapy for intermediate-risk prostate cancer (PCa) remains under debate because strategies with or without local therapy, such as radical prostatectomy (RP) or radiotherapy (RT), can be reasonably recommended according to major urology guidelines [1–3]. While both RP and RT are effective in delaying disease progression and reducing premature death rates in intermediate-risk PCa, treatment toxicity may sometimes outweigh the oncological benefits [2, 4–8]. In consequence, clinical decision-making concerning local therapy may be most complex in intermediate-risk PCa patients. Conventional overall or cancer-specific survival metrics report whether and how often death occurs in relation to the timepoint of treatment / diagnosis, but not when in a patient’s remaining lifespan it occurs, although the age of death has substantial implications on treatment counseling and the societal burden. This limitation is particularly consequential in intermediate-risk PCa, where cancer-related deaths are comparatively rare, accrue over follow-up periods of one to two decades, and compete with substantial background mortality from cardiovascular and other age-related conditions. To overcome these shortcomings, a comparison of PCa burden measured in average years of life lost (YLL) between different initial local therapy strategies may be most beneficial in intermediate-risk PCa patients. However, such comparisons have not been performed.

To address this knowledge gap, we designed and completed a comparison between RP, RT, and no local therapy patients using a controlled framework with Social Security Administration (SSA) life tables simulated population-based controls. We relied on the established YLL metric [9] to estimate the differences between the actual survival of intermediate-risk PCa patients relative to Monte Carlo simulated population-based controls life expectancy. We hypothesized that YLL, as a measure of premature death, would not differ between intermediate-risk PCa patients treated with RP, RT, or no local therapy. We tested this hypothesis in a contemporary, large-scale cohort of intermediate-risk PCa patients within the Surveillance, Epidemiology, and End Results (SEER) database (2004–2021).

## Materials and methods

### Study population

Within the SEER database 2004–2021, we identified individuals aged 45–75 and diagnosed with histologically confirmed intermediate-risk PCa. Patients with metastatic (cN1, *n* = 17,854 or cM1, *n* = 7,295) or low-/high-risk PCa (*n* = 296,149) were excluded. Moreover, patients were excluded if age at diagnosis, year of diagnosis, clinical stage, and vital status were missing (*n* = 238,248, Supplementary Fig. 1). In accordance with the NCCN Clinical Practice Guidelines in Oncology (NCCN Guidelines^®^) [1, 10], intermediate-risk PCa was defined as the absence of (very) high-risk features (i.e., ≥cT3, ISUP grade group ≥ 4, PSA ≥ 20 ng/ml) and the presence of at least one intermediate-risk factor (IRF): clinical stage cT2b-c, ISUP grade group 2–3, or PSA 10–20 ng/ml. Favorable intermediate-risk was defined as one IRF, ISUP grade group 1–2, and < 50% positive biopsy cores. Unfavorable intermediate-risk was defined as more than one IRF, ISUP grade group 3, and/or ≥ 50% positive biopsy cores. The percentages of positive biopsy cores were not available for patients diagnosed before 2010. Patients were stratified according to local therapy: radical prostatectomy vs. radiotherapy (consisting of external beam radiation therapy, brachytherapy alone, brachytherapy plus external beam) vs. no local therapy (including non-radical therapies like transurethral resection of the prostate).

### Statistical analyses

Preregistration was obtained prior to analyzing the data (doi:10.17605 - OSF.IO/D2RUV). First, we identified intermediate-risk PCa patients in the SEER database (2004–2021). Subsequently, a Monte Carlo simulation was used to generate simulated controls in a one-to-one fashion, which accurately reflected the age, sex, and inclusion year of each PCa patient. For each simulated control, a Markov chain representing natural age progression was constructed based on SSA life Tables (2004–2021) [11]. This approach follows established methodology [12, 13] and is endorsed by the NCCN Clinical Practice Guidelines in Oncology (NCCN Guidelines^®^) [1]. The follow-up of the simulated controls reflected the actual follow-up of SEER cases according to the year of diagnosis. For example, patients diagnosed in 2011 had a median follow-up period of 124 months. Therefore, the corresponding simulated controls for PCa patients diagnosed in 2011 could either survive or die within each of the 124 simulated month intervals, according to the SSA life table probabilities. Thus derived life expectancy of simulated controls and the actual survival of PCa patients were graphically depicted using Kaplan-Meier plots. The log-rank test was applied for survival comparisons between PCa patients and controls. Based on Kaplan-Meier estimates, average years of life lost (YLL) were estimated as the restricted mean survival time between the age at death of PCa patients and the remaining life expectancy of population-based controls until the age of 75:


5


The age cut-off of 75 was used in accordance with the definition of premature death by the National Cancer Institute [14]. Subgroup analyses were performed according to the age at diagnosis (45–55 vs. 56–65 vs. 66–75) and intermediate-risk subsets (favorable vs. unfavorable). Continuous variables were compared using the Wilcoxon rank-sum test and categorical variables using Pearson’s chi-squared test. The R software environment was used for statistical computing and graphics (R version 4.4.1 (2024-06-14), The R Foundation for Statistical Computing, Vienna, Austria). All tests were two-sided with a significance level of *p* < 0.05 without adjustment to multiple testing (Table 1).

## Results

### Overall characteristics

Of 220,113 intermediate-risk PCa patients, 94,452 (42.9%), 85,232 (38.7%), and 40,429 (18.4%) underwent RP, RT, or no local therapy, respectively. Compared with RT or no local therapy patients, RP patients were diagnosed at a younger age (62, IQR 57–66 vs. 67, IQR 62–71 or 66, IQR 61–71 years) and exhibited lower PSA values (6.0, IQR 4.7–8.6 vs. 7.0, IQR 5.2–10.4 or 7.9, IQR 5.4–11.6 ng/ml). RP and RT patients more frequently exhibited ISUP grade group > 1 (88.9 and 87.4 vs. 71.7%) and clinical stage T2b-c (15.4 and 11.4 vs. 8.6%) than those without local therapy (all *p* < 0.001).

### Years of life lost in radical prostatectomy patients

The estimation of YLL in RP patients rested on 94,452 actual patients and 94,452 population controls. Premature death was recorded in 6,979 (7.4%) patients and 12,918 (13.7%) population controls (Supplementary Fig. 2, *p* < 0.001). On average, RP patients outlived their population controls by 0.69 years (Table 2).

**Table 1:** Descriptive characteristics of intermediate-risk prostate cancer patients, stratified by local therapy. (*n* = 220,113)

	Radical prostatectomy (*n*=94,*452*,* 42.9%)*	Radiotherapy (*n*=85,*232*,* 38.7%)*	No local therapy (*n*=40,*429*,*18.4%)*	*p*-value^1^
Age at diagnosis, median (IQR)	62 (57, 66)	67 (62, 71)	66 (61, 71)	<0.001
45 – 55	18,172 (19.2%)	5,415 (6.4%)	3,283 (8.1%)	<0.001
56 – 65	47,496 (50.3%)	29,814 (35.0%)	14,932 (36.9%)	
66 – 75	28,784 (30.5%)	50,003 (58.7%)	22,214 (54.9%)	
*Intermediate subgroup*, *n (%)*				<0.001
Favorable	51,394 (54.4%)	40,383 (47.4%)	25,416 (62.9%)	
Unfavorable	43,058 (45.6%)	44,849 (52.6%)	15,013 (37.1%)	
*PSA,* *median (IQR)*	6.0 (4.7, 8.6)	7.0 (5.2, 10.4)	7.9 (5.4, 11.6)	<0.001
*ISUP*, *n (%)*				<0.001
1	10,476 (11.1%)	10,764 (12.6%)	11,445 (28.3%)	
2	61,919 (65.6%)	50,079 (58.8%)	21,795 (53.9%)	
3	22,057 (23.4%)	24,389 (28.6%)	7,189 (17.8%)	
*Clinical stage,* *n (%)*				<0.001
≤ T2a	79,874 (84.6%)	75,479 (88.6%)	36,935 (91.4%)	
T2b-c	14,578 (15.4%)	9,753 (11.4%)	3,494 (8.6%)	

^1^Wilcoxon rank-sum test, Pearson´s chi-squared test. The definition of intermediate-risk prostate cancer as well as the favorable/unfavorable subsets relied on the NCCN Clinical Practice Guidelines in Oncology (NCCN Guidelines^®^) for Prostate Cancer V.1.2025. © 2025 National Comprehensive Cancer Network, Inc. All rights reserved

*PSA* prostate specific antigen, *IQR* Interquartile range, *ISUP* International Society of Urological Pathology

**Table 2:** Years of life lost (-) or gained (+) in intermediate-risk prostate cancer patients, stratified by local therapy and patient characteristics

	Radical prostatectomy (*n*=94,*452*,* 42.9%)*	Radiotherapy (*n*=85,*232*,* 38.7%)*	No local therapy (*n*=40,*429*,*18.4%)*
Overall	+0.69*	+0.06*	–0.21*
*Age at diagnosis*			
45 – 55	+0.78*	-0.69*	–1.32*
56 – 65	+0.79*	-0.06*	–0.49*
66 – 75	+0.33*	+0.11*	–0.04*
*Intermediate-risk subsets*			
Favorable	+0.80*	+0.11*	–0.05*
Unfavorable	+0.56*	+0.01*	–0.47*

Years of life lost (-) or gained (+) are Kaplan-Meier derived averages in relation to life expectancy predictions up to 75 years from Social Security Administration (SSA) life tables. A positive value indicates a survival benefit in relation to SSA controls. A negative value indicates shorter survival of SEER cases in relation to SSA controls (= actual years of life lost). Predicted survival were based on Monte Carlo simulation based on SSA life tables. The definition of intermediate-risk prostate cancer as well as the favorable/unfavorable subsets relied on the NCCN Clinical Practice Guidelines in Oncology (NCCN Guidelines^®^) for Prostate Cancer V.1.2025. © 2025 National Comprehensive Cancer Network, Inc. All rights reserved. * log-rank *p*<0.0001

In subgroup analyses according to age intervals at diagnosis, RP patients outlived the population controls by 0.78, 0.79, and 0.33 years, when diagnosed at ages 45–55, 56–65, and 66–75, respectively. In subgroup analyses of favorable vs. unfavorable intermediate-risk PCa, RP patients outlived the population controls by 0.80 vs. 0.56 years, respectively.

### Years of life lost in radiotherapy patients

The estimation of YLL in RT patients rested on 85,232 actual patients and 85,232 population controls. Premature death was recorded in 8,193 (9.6%) patients and 9,101 (10.7%) population controls (Supplementary Fig. 2, *p* < 0.001). On average, RT patients outlived population controls by 0.06 years (Table 2).

In subgroup analyses according to age intervals at diagnosis, RT patients diagnosed at age 45–55 and 56–65 lost 0.69 and 0.06 years of life, respectively. Conversely, RT patients diagnosed at the age of 66–75 outlived population controls by 0.11 years. This apparent survival advantage of older RT patients most likely reflects the selection of patients with favorable overall health for curative RT at an advanced age, rather than a protective treatment effect. In subgroup analyses of favorable vs. unfavorable intermediate-risk PCa, RT patients outlived population controls by 0.11 vs. 0.01 years, respectively.

### Years of life lost in patients without local therapy

The estimation of YLL in patients without local therapy rested on 40,429 actual patients and 40,429 population controls. Premature death was recorded in 4,350 (10.8%) patients and 3,705 (9.2%) population controls (Supplementary Fig. 2, *p* < 0.001). On average, no local therapy patients exhibited a survival disadvantage of 0.21 YLL, compared to population controls (Table 2).

In subgroup analyses according to age intervals at diagnosis, no local therapy patients lost 1.32, 0.49, and 0.04 years of life, when diagnosed at age 45–55, 56–65, and 66–75, respectively. Among patients without local therapy, those with favorable intermediate-risk PCa exhibited a survival disadvantage of 0.05 YLL, whereas those with unfavorable intermediate-risk PCa exhibited a survival disadvantage of 0.47 YLL (Table 2).

## Discussion

The optimal initial local therapy for intermediate-risk prostate cancer (PCa) remains still under debate. We hypothesized that we will not capture YLL differences within controlled comparisons of different initial local therapy strategies. Although our findings are in perfect agreement with previous studies focusing on overall and cancer-specific mortality, the current analysis revealed several noteworthy findings.

First, the total number of deaths or survival years after different initial local therapy strategies does not provide a good metric for quantifying differences in the burden of disease because such measures assign the same weight to a death at age 75 as they do at age 55. Years of life lost (YLL) represents an established measure of premature mortality that considers both the frequency of death and the age at which it occurs, in relation to life expectancy. The YLL metric has repeatedly been used in official documentation by health agencies such as the International Agency for Research on Cancer of the World Health Organization [15, 16] and the National Cancer Institute [17]. YLL was a key measure of the Global Burden of Disease Study [18]. Moreover, YLL has been applied to quantify the numerical extent to which PCa had prematurely curtailed survival within entire populations [19–21]. However, no such report was able to assess the differences in PCa characteristics, initial local therapy strategies, or age at diagnosis in relation to YLL [22, 23]. Such differentiation is crucial in PCa, which exhibits high heterogeneity regarding cancer control outcomes and disproportionally affects the elderly population. In particular, the complexity of initial local therapy decision-making may be more elevated in intermediate-risk PCa than in low- or high-risk PCa patients. To circumvent these limitations, we used a novel approach to quantify YLL, that may distinguish intermediate-risk PCa patients with initial local therapy from those who have not benefited from local therapy. YLL had not been exploited in intermediate-risk PCa before but does represent a meaningful measure for clinical decision-making and patient counseling.

Second, the current analysis rested on controlled comparisons. Specifically, the life expectancy of controls were based on the official sex-specific U.S. SSA life tables using Markov chain survival simulations [11]. The SSA life tables provide key actuarial data to calculate life expectancy in an unbiased fashion. As a proof of its robust estimation capabilities, the derived life expectancy averages are universally used for social security trust fund management, pension calculations, and life insurance planning. Moreover, SSA life tables have officially been endorsed for life expectancy estimation in PCa patients according to the NCCN Clinical Practice Guidelines in Oncology (NCCN Guidelines^®^) [1] and have been extensively validated for this explicit purpose [24]. The National Cancer Institute recommends the use of life tables for YLL calculations as well [17]. We further relied on the premature death definition of 75 years by the National Cancer Institute, which was used in previous analyses [14] and has been endorsed by other official agencies as well [25].

Third, the above steps allowed us to quantify and compare YLL between intermediate-risk PCa patients and population controls. For locally treated patients, the YLL numbers indicated that RP and RT patients outlived population controls by 0.69 and 0.06 years, respectively. Conversely, no local therapy patients exhibited a survival disadvantage quantified by 0.21 YLL, relative to population-based controls. In consequence, YLL disadvantages distinguished no local therapy patients from their RP or RT counterparts. These observations, that are based for the first time on the YLL methodology, are in perfect agreement with previous reports that relied on other metrics [5, 24]. This represents an internal validation of the methodology and data used in the current study.

Fourth, our methodology provided specific YLL estimates and differences for groups of particular interest based on patient and cancer characteristics. Specifically, no YLL disadvantages were observed in RP patients, irrespective of the age interval at diagnosis stratification. In contrast, substantial higher YLL values were observed in no local therapy patients diagnosed at age 45–55 compared to those in no local therapy patients diagnosed at 66–75 years (1.32 vs. 0.04 YLL). These differences agree with previous reports and clinical practice that young patients especially benefit from RP [26–29]. For example, in a landmark study by Bill-Axelson, radical prostatectomy reduced the absolute overall mortality risk by 25.5 vs. 1.9% in patients aged < 65 and ≥ 65, respectively [5]. Moreover, patients diagnosed and treated at an advanced age, such as 66–75, have less years to be at risk for premature death. Therefore, the YLL differences between therapies are less pronounced when the age at diagnosis approaches the definition of premature death.

Fifth, we further stratified the patients into favorable and unfavorable intermediate-risk PCa subsets. No YLL disadvantages were observed in RP and RT patients, irrespective of intermediate-risk subsets. Conversely, patients without local therapy exhibited a higher YLL in the unfavorable subset than in the favorable subset (0.47 vs. 0.05). These findings are in perfect agreement with previous reports [30–32] and the actual NCCN Clinical Practice Guidelines in Oncology (NCCN Guidelines^®^), which recommend no local therapy strategies, such as active surveillance, for favorable, but not unfavorable, intermediate-risk PCa patients [1].

Taken together, the current study used a novel framework to estimate YLL differences in intermediate-risk PCa patients. No YLL disadvantage was observed in locally treated intermediate-risk PCa patients. Conversely, patients who did not receive local therapy exhibited a survival disadvantage of 0.21 YLL. In subgroup analyses, the highest YLL was observed in no local therapy patients diagnosed at a young age and with unfavorable intermediate-risk PCa. Therefore, these patients represent targets for which no local therapy strategies should be avoided.

Although a large-scale analysis required the use of retrospective, population-based databases, the current study had inherent limitations that relied on this approach. First, it shares the limitations of all similar studies that were based on SEER databases and used a retrospective data design. In detail, percentages of positive biopsy were not available before 2010. Second, the SEER database did not capture detailed information on why no local treatment was administered. Therefore, it is not possible to differentiate between the effects of local therapy itself and eligibility for local therapy. Third, information on comorbidities and exact disease extent were not available, which might have influenced treatment decisions and life expectancy as well. While SSA controls provide robust averages for health economics and outcomes research, clinical interpretations should be drawn with some caution. However, other nomograms, such as the Cowen or Tewari nomograms [33, 34], require further information, such as body mass index, Charlson comorbidity index, or performance status, which are not available within the SEER or other large-scale databases. Fourth, survival-only based metrics such as YLL do not capture the metastatic burden carried by initially untreated patients, that – as demonstrated in the ProtecT trial [7] – may not translate into a survival difference even after 15 years of follow-up, but profoundly affects subsequent systemic therapy, palliative interventions, cost-of-care, and quality-of-life .

## Supplementary Information

Below is the link to the electronic supplementary material.

Supplementary Material 1

Supplementary Material 2

## Data Availability

All data supporting the findings of this study are available within the SEER program.

## Declarations

The institutional review board waived study-specific ethics approval owing to the anonymous design of the SEER database.

### Registry and the Registration No. of the study/trial

Registration was obtained prior to the analysis of the data: Falkenbach, Fabian. 2025. “The Effect of Local Treatment on Years of Life Lost in Localized Prostate Cancer.” OSF. January 31. doi:https://doi.org/10.17605/OSF.IO/D2RUV.

## References

[1] National Comprehensive Cancer Network, Inc (2025) NCCN Clinical Practice Guidelines in Oncology (NCCN Guidelines^®^) for Prostate Cancer V.1.2025

[2] Cornford P, van den Bergh RCN, Briers E, Van den Broeck T, Brunckhorst O, Darraugh J et al EAU-EANM-ESTRO-ESUR-ISUP-SIOG Guidelines on Prostate Cancer-2024 Update. Part I: Screening, Diagnosis, and Local Treatment with Curative Intent. Eur Urol 2024:S0302-2838(24)02254-1. 10.1016/j.eururo.2024.03.027 38614820

[3] Eastham JA, Auffenberg GB, Barocas DA, Chou R, Crispino T, Davis JW et al (2022) Clinically Localized Prostate Cancer: AUA/ASTRO Guideline, Part I: Introduction, Risk Assessment, Staging, and Risk-Based Management. J Urol 208:10–18. 10.1097/JU.0000000000002757 35536144

[4] Eastham JA, Auffenberg GB, Barocas DA, Chou R, Crispino T, Davis JW et al (2022) Clinically Localized Prostate Cancer: AUA/ASTRO Guideline, Part II: Principles of Active Surveillance, Principles of Surgery, and Follow-Up. J Urol 208:19–25. 10.1097/JU.0000000000002758 35536148

[5] Bill-Axelson A, Holmberg L, Garmo H, Rider JR, Taari K, Busch C et al (2014) Radical prostatectomy or watchful waiting in early prostate cancer. N Engl J Med 370:932–942. 10.1056/NEJMoa1311593 PMC4118145 24597866

[6] Wilt TJ, Jones KM, Barry MJ, Andriole GL, Culkin D, Wheeler T et al (2017) Follow-up of Prostatectomy versus Observation for Early Prostate Cancer. N Engl J Med 377:132–142. 10.1056/NEJMoa1615869 28700844

[7] Hamdy FC, Donovan JL, Lane JA, Metcalfe C, Davis M, Turner EL et al (2023) Fifteen-Year Outcomes after Monitoring, Surgery, or Radiotherapy for Prostate Cancer. N Engl J Med 388:1547–1558. 10.1056/NEJMoa2214122 36912538

[8] Donovan JL, Hamdy FC, Lane JA, Young GJ, Metcalfe C, Walsh EI et al (2023) Patient-Reported Outcomes 12 Years after Localized Prostate Cancer Treatment. NEJM Evid 2:EVIDoa2300018. 10.1056/EVIDoa2300018 38320051

[9] Martinez R, Soliz P, Caixeta R, Ordunez P (2019) Reflection on modern methods: years of life lost due to premature mortality-a versatile and comprehensive measure for monitoring non-communicable disease mortality. Int J Epidemiol 48:1367–1376. 10.1093/ije/dyy254 PMC6693813 30629192

[10] Longoni M, Di Bello F, Rodriguez Peñaranda N, Falkenbach F, Marmiroli A, Le QC et al Clinically Meaningful Upstaging and Upgrading in Favorable Intermediate-Risk Radical Prostatectomy Patients. Clin Genitourin Cancer 2025:102300. 10.1016/j.clgc.2025.102300 39863496

[11] Social Security Administration (2024) https://www.ssa.gov/oact/STATS/table4c6.html (accessed February 1, 2025)

[12] Falkenbach F, Le QC, Longoni M, Marmiroli A, Catanzaro C, Nicolazzini M et al (2025) Years of Life Lost in Metastatic and Locally Advanced Prostate Cancer. Eur Urol Oncol 8:961–967. 10.1016/j.euo.2025.04.008 40675872

[13] Quarta L, Nicolazzini M, Catanzaro C, Polverino F, Petix M, Filzmayer M et al (2026) Years of life lost and mortality rates from metastatic prostate cancer in contemporary African Americans vs. Caucasians. World J Urol 44:189. 10.1007/s00345-026-06295-3 41729247

[14] Definition of premature death - NCI Dictionary of Cancer Terms - NCI (2011) https://www.cancer.gov/publications/dictionaries/cancer-terms/def/premature-death (accessed January 22, 2025)

[15] World HO (2025) accessed February 5,. Years of life lost from mortality (YLL). Global Health Observatory 2024. https://www.who.int/data/gho/indicator-metadata-registry/imr-details/4427

[16] Rumgay H, Cabasag CJ, Offman J, de Camargo Cancela M, Barchuk A, Mathur P et al (2023) International burden of cancer deaths and years of life lost from cancer attributable to four major risk factors: a population-based study in Brazil, Russia, India, China, South Africa, the United Kingdom, and United States. EClinicalMedicine 66:102289. 10.1016/j.eclinm.2023.102289 PMC10772154 38192589

[17] National Cancer Institute, NIH, Cancer Trends Progress (2024) Report https://progressreport.cancer.gov/end/life_lost (accessed February 5, 2025)

[18] Fitzmaurice C, Allen C, Barber RM, Barregard L, Bhutta ZA, Brenner H et al (2017) Global, Regional, and National Cancer Incidence, Mortality, Years of Life Lost, Years Lived With Disability, and Disability-Adjusted Life-years for 32 Cancer Groups, 1990 to 2015: A Systematic Analysis for the Global Burden of Disease Study. JAMA Oncol 3:524–548. 10.1001/jamaoncol.2016.5688 PMC6103527 27918777

[19] Withrow D, Pilleron S, Nikita N, Ferlay J, Sharma S, Nicholson B et al (2022) Current and projected number of years of life lost due to prostate cancer: A global study. Prostate 82:1088–1097. 10.1002/pros.24360 PMC9246888 35468227

[20] Launer BM, Lloyd GL (2021) Sociodemographic index and global trends in prostate cancer: 1990–2017. Prostate 81:825–831. 10.1002/pros.24178 34227144

[21] Guzman-Esquivel J, Murillo-Zamora E, Ortiz-Mesina M, Galvan-Salazar HR, De-Leon-Zaragoza L, Casarez-Price JC et al (2023) Regional and national burden of prostate cancer: incidence, mortality, years of life lost, and disability-adjusted life years, in Mexico and Latin America from 1990 to 2019. Int Urol Nephrol 55:2155–2160. 10.1007/s11255-023-03653-7 PMC10240458 37273013

[22] Gwalani P, GOR D, Shah A, Thakur RK, Gartrell BA (2024) The silent burden: A SEER-based analysis of potential years of life lost due to genitourinary cancers in the United States (1975–2017). J Clin Oncol. 10.1200/JCO.2024.42.16_suppl.e17107

[23] Kamel MH, Bimali M, Khalil MI, Eltahawy E, Su Lj, Bissada NK et al (2019) Regional trends in average years of potential life lost (AYPLL) secondary to prostate cancer deaths among Caucasians and African Americans treated by surgery or radiation. Int Urol Nephrol 51:561–569. 10.1007/s11255-019-02116-2 30840195

[24] Preisser F, Bandini M, Mazzone E, Nazzani S, Marchioni M, Tian Z et al (2019) Validation of the Social Security Administration Life Tables (2004–2014) in Localized Prostate Cancer Patients within the Surveillance, Epidemiology, and End Results database. Eur Urol Focus 5:807–814. 10.1016/j.euf.2018.05.006 29802052

[25] Statistics Canada. Premature mortality 2014. https://www23.statcan.gc.ca/imdb/p3VD.pl?Function=getVD&TVD=151618&CVD=151619&CLV=0&MLV=3&D=1(accessed February 5, 2025).

[26] Mehring G, Tilki D, Heinzer H, Steuber T, Pose RM, Thederan I et al (2023) Histopathological results of radical prostatectomy specimen of men younger than 50 years of age at the time of surgery: possible implications for prostate cancer screening programs? World J Urol 41:421–425. 10.1007/s00345-023-04287-1 PMC9947052 36656332

[27] Becker A, Tennstedt P, Hansen J, Trinh QD, Kluth L, Atassi N et al (2014) Functional and oncological outcomes of patients aged < 50 years treated with radical prostatectomy for localised prostate cancer in a European population. BJU Int 114:38–45. 10.1111/bju.12407 24053677

[28] Tilki D, Maurer V, Pompe RS, Chun FK, Preisser F, Haese A et al (2020) Tumor characteristics, oncological and functional outcomes after radical prostatectomy in very young men ≤ 45 years of age. World J Urol 38:95–101. 10.1007/s00345-019-02740-8 30937571

[29] Salinas CA, Tsodikov A, Ishak-Howard M, Cooney KA (2014) Prostate cancer in young men: an important clinical entity. Nat Rev Urol 11:317–323. 10.1038/nrurol.2014.91 PMC4191828 24818853

[30] Zumsteg ZS, Spratt DE, Pei I, Zhang Z, Yamada Y, Kollmeier M et al (2013) A new risk classification system for therapeutic decision making with intermediate-risk prostate cancer patients undergoing dose-escalated external-beam radiation therapy. Eur Urol 64:895–902. 10.1016/j.eururo.2013.03.033 23541457

[31] Tosoian JJ, Mamawala M, Epstein JI, Landis P, Wolf S, Trock BJ et al (2015) Intermediate and Longer-Term Outcomes From a Prospective Active-Surveillance Program for Favorable-Risk Prostate Cancer. J Clin Oncol 33:3379–3385. 10.1200/JCO.2015.62.5764 PMC4863946 26324359

[32] Simpkin AJ, Tilling K, Martin RM, Lane JA, Hamdy FC, Holmberg L et al (2015) Systematic Review and Meta-analysis of Factors Determining Change to Radical Treatment in Active Surveillance for Localized Prostate Cancer. Eur Urol 67:993–1005. 10.1016/j.eururo.2015.01.004 25616709

[33] Cowen ME, Halasyamani LK, Kattan MW (2006) Predicting life expectancy in men with clinically localized prostate cancer. J Urol 175:99–103. 10.1016/S0022-5347(05)00018-2 16406881

[34] Tewari A, Johnson CC, Divine G, Crawford ED, Gamito EJ, Demers R et al (2004) Long-term survival probability in men with clinically localized prostate cancer: a case-control, propensity modeling study stratified by race, age, treatment and comorbidities. J Urol 171:1513–1519. 10.1097/01.ju.0000117975.40782.95 15017210

