# Supplementary material for: Local treatment vs. years of life lost in intermediate-risk prostate cancer
Source: World J Urol. 2026 Oct 30;44(1):774. doi: 10.1007/s00345-026-06858-4 (PMC13722718; doi:10.1007/s00345-026-06858-4)
Supplement: Supplementary file 1 [file 345_2026_6858_MOESM1_ESM.pdf]

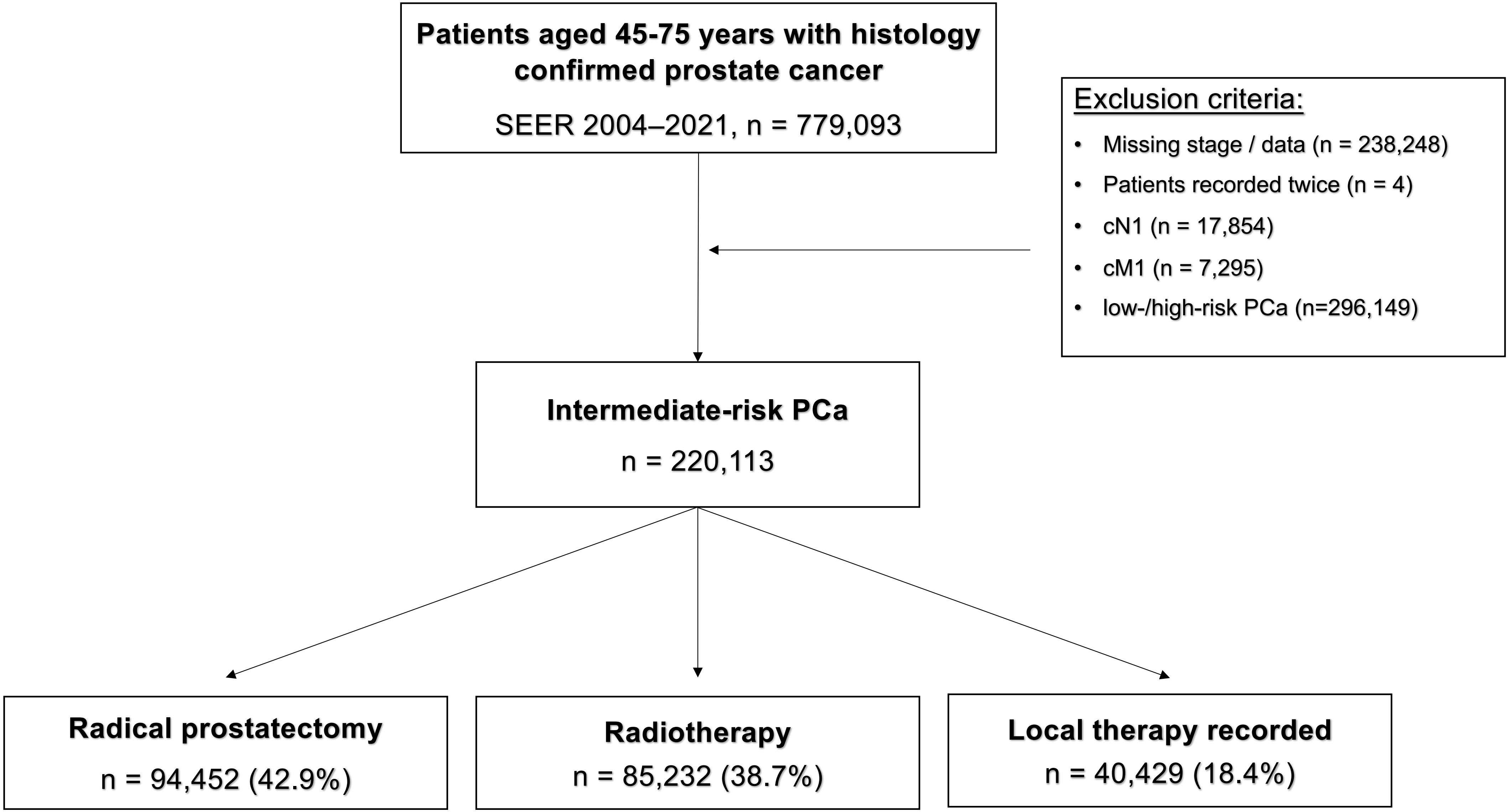

Supplementary Figure 1: Patient selection
