# Supplementary material for: Local treatment vs. years of life lost in intermediate-risk prostate cancer
Source: World J Urol. 2026 Oct 30;44(1):774. doi: 10.1007/s00345-026-06858-4 (PMC13722718; doi:10.1007/s00345-026-06858-4)
Supplement: Supplementary file 2 [file 345_2026_6858_MOESM2_ESM.pdf]

Supplementary Figure 2: Kaplan-Meier plots depicting observed and predicted survival for intermediate-risk prostate cancer patients, stratified by local therapy.

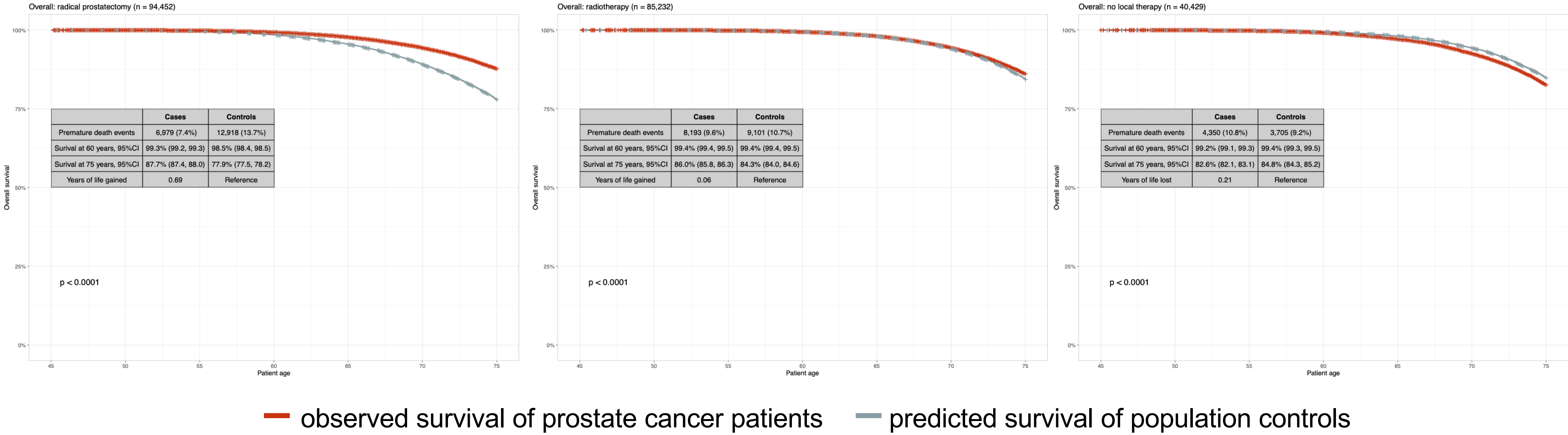

Overall: radical prostatectomy (n = 94,452)

	Cases	Controls
Premature death events	6,979 (7.4%)	12,918 (13.7%)
Surival at 60 years, 95%CI	99.3% (99.2, 99.3)	98.5% (98.4, 98.5)
Surival at 75 years, 95%CI	87.7% (87.4, 88.0)	77.9% (77.5, 78.2)
Years of life gained	0.69	Reference

Overall: radiotherapy (n = 85,232)

	Cases	Controls
Premature death events	8,193 (9.6%)	9,101 (10.7%)
Surival at 60 years, 95%CI	99.4% (99.4, 99.5)	99.4% (99.4, 99.5)
Surival at 75 years, 95%CI	86.0% (85.8, 86.3)	84.3% (84.0, 84.6)
Years of life gained	0.06	Reference

Overall: no local therapy (n = 40,429)

	Cases	Controls
Premature death events	4,350 (10.8%)	3,705 (9.2%)
Surival at 60 years, 95%CI	99.2% (99.1, 99.3)	99.4% (99.3, 99.5)
Surival at 75 years, 95%CI	82.6% (82.1, 83.1)	84.8% (84.3, 85.2)
Years of life lost	0.21	Reference
